# *Lavandula pedunculata* subsp. *atlantica*: A Multifunctional Essential Oil for Potentially Combating Microbial Infections and Inflammatory Processes

**DOI:** 10.3390/molecules30112267

**Published:** 2025-05-22

**Authors:** Giusy Castagliuolo, Natale Badalamenti, Vincenzo Ilardi, Gianfranco Fontana, Dario Antonini, Mario Varcamonti, Maurizio Bruno, Anna Zanfardino

**Affiliations:** 1 Department of Biology, University of Naples Federico II, 80126 Naples, Italy; giusy.castagliuolo@unina.it (G.C.); dario.antonini@unina.it (D.A.); mario.varcamonti@unina.it (M.V.); anna.zanfardino@unina.it (A.Z.); 2 Department of Biological, Chemical and Pharmaceutical Sciences and Technologies (STEBICEF), Università degli Studi di Palermo, Viale delle Scienze, Ed. 17, 90128 Palermo, Italy; natale.badalamenti@unipa.it (N.B.); vincenzo.ilardi@unipa.it (V.I.); maurizio.bruno@unipa.it (M.B.); 3 NBFC, National Biodiversity Future Center, 90133 Palermo, Italy

**Keywords:** *Lavandula pedunculata* subsp. *atlantica*, Lamiaceae, essential oil, fenchone, antimicrobial, antibiofilm

## Abstract

The genus *Lavandula* L., belonging to the Lamiaceae family, contains about forty species with a distribution that mainly extends in the Mediterranean basin from the North Atlantic to the Middle East. Due to their excellent biological properties, the aerial parts and flowers of *Lavandula peduncolata* ssp. have been utilized in traditional medicine in Morocco and western Europe. This paper investigated the chemical composition and antibacterial activities as well as the antibiofilm and antioxidant activities of the essential oil (EO) obtained from the pre-flowering aerial parts of *Lavandula pedunculata* subsp. *atlantica* collected in Morocco. The chemical composition of the EO, obtained by classic hydrodistillation, showed by GC-MS analysis the presence of a large amount of oxygenated monoterpene compounds. The main constituents of the EO were camphor (27.8%), camphene (10.9%), fenchone (10.6%), and eucalyptol (8.5%). The EO has been evaluated for its antimicrobial, antibiofilm, antioxidant and anti-inflammatory properties, showing promising activity against both Gram-positive and Gram-negative strains. These findings highlight the potential of *Lavandula* EO in combating infections caused by *Streptococcus mutans* and *Streptococcus oralis* (oral diseases), *Staphylococcus aureus* (skin infections), *Escherichia coli* and *Shigella sonnei* (gastrointestinal and urinary infections). In addition, although the EO showed no evident effects on cell viability in eukaryotic epithelial cells, it exhibited promising effects on anti-inflammatory properties.

## 1. Introduction

The genus *Lavandula* L., belonging to the Nepetoideae subfamily of the Lamiaceae family, is mainly distributed in the Mediterranean basin from the North Atlantic to the Middle East. Plants of this family are biennial herbaceous which mainly occur on dry, sunny, calcareous or siliceous soils. According to the excellent taxonomic description of the genus *Lavandula* of Upson and Andrews [1], it consists of around forty species and eighty intraspecific taxa and hybrids. They are divided into three subgenera (i.e., *Fabricia*, *Sabaudia*, and *Lavandula*) and eight sections. Several species are cultivated for commercial purposes such as fine lavender (*L. angustifolia* Mill.), lavender aspic (*L. latifolia* Medik.), woolly lavender (*L. lanata* Boiss.), and lavandin (*L. x intermedia* Emeric ex Loisel., a sterile hybrid of *L. angustifolia* and *L. latifolia*), all belonging to the Lavandula section and the *Lavandula* subgenus [2].

*Lavandula pedunculata* (Mill.) Cav. (syn: *L. stoechas* subsp. *pedunculata* (Mill.) Rozeira; *Stoechas pedunculata* Mill.) is a subshrub growing primarily in the temperate biome, native to Madeira, Iberian Peninsula to Morocco, East Aegean Islands to northwest and west Turkey [3]. It includes six accepted infraspecifics subspecies: *L. pedunculata* subsp. *cariensis* (Boiss.) Upson & S.Andrews, native to the East Aegean Islands and Turkey, *L. pedunculata* subsp. *lusitanica* (Chaytor) Franco and *L. pedunculata* subsp. *sampaioana* (Rozeira) Franco, both present only in the Iberian Peninsula, *L. pedunculata* subsp. *maderensis* (Benth.) Menezes, endemic of Madeira Island, *L. pedunculata* subsp. *pedunculata* present in Morocco, Spain and Portugal, and *L. pedunculata* subsp. *atlantica* (Braun-Blanq.) Romo, which is endemic of Morocco.

**Figure 1:**
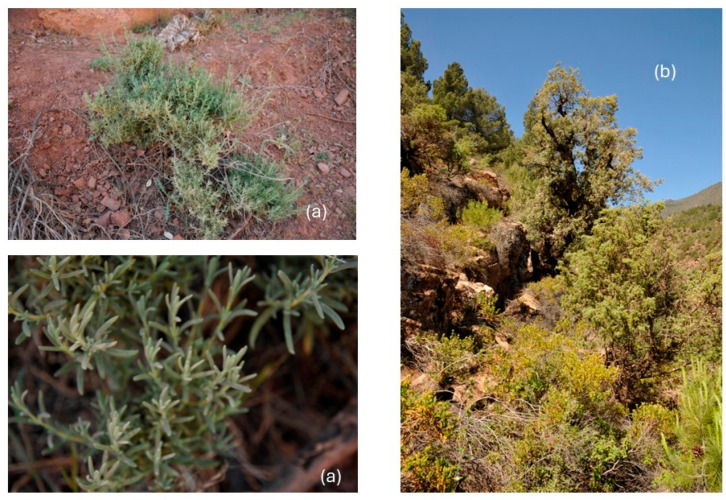
The habitus of *Lavandula pedunculata* subsp. *atlantica* (**a**); pre-flowering aerial parts (**b**); the rocky habitats at Ait Ben Ammar (Morocco).

*L. pedunculata* subsp. *atlantica* (Braun-Blanq.) Romo [syn. *L. atlantica* (Braun-Blanq.) Braun-Blanq. & Maire, *L. stoechas* subsp. *atlantica* Braun-Blanq., *L. pedunculata* f. *brevipedunculata* Caball.)] (Figure 1) is a small shrub with grayish-green, aromatic leaves that are simple, entire, lanceolate, and 3–4 cm long. The fertile bracts are circular, broadly obovate and rounded, whereas the sterile ones are brief. The inflorescences are 3–4 cm long with blue–purple flowers carried by a peduncle (3–10 cm). This taxon, native to the Atlas Mountains of Morocco, grows on subhumid clay–silt at shallow depth, with a high load of shale–sandstone, and it is rather uncommon in cultivation [4].

Several ethnopharmacological uses have been reported for *L. pedunculata*. It is known in Morocco with several vernacular names: *halhal*, *izri*, *amezzir*, *timerza*, *khzama*, *muqef rwah*, *chelchel*, etc. [5]. The infusion of the flowers and leaves of *L. pedunculata*, known as “Khzemt/خزمامث”, is used in Morocco as cataplasm for skin disease [6]. The decoction of the leaves of the same species is used in the Rif, Morocco (vernacular name *lakhzama*) for lung cancer [7]. The leaves and inflorescences are also used as tea, oral ingestion, poultices or washes for dermatological, general health, gastrointestinal, gynecological, ophthalmological, otolaryngological, respiratory and pediatric (*kolshi*, *azbar*, *atsirid*, *ch’aar*, *meda*) troubles [8].

In Portugal, the aerial parts and flowers of *L. pedunculata* (Mill.) Cav., where it is known as *rosmaninho*, *arçã*, are used in the popular medicine for digestion, headaches, asthma and bronchitis showing therapeutic properties such as anti-asthmatic, anti-migraine, bronchodilator, and stomachic [9]. In the same country, it is the flowered aerial parts are utilized in folk medicine as infusions, for anxiety and insomnia, as digestive and as a therapeutic agent with antiseptic action for cleaning wounds [10,11]. Active metabolites in the plant have a positive effect on human health [12], and decoctions have acetylcholinesterase inhibitory capacity [11].

Several investigations have been published on the composition of the essential oil (EO) of *L. pedunculata* taxa (Table 1) [13,14,15,16,17,18,19,20,21,22,23,24] also with the aim to determine their biological properties. The antimicrobial activity of several *Lavandula* species has been largely confirmed both on Gram-negative and Gram-positive bacteria [13,16] as well as against bacteria and fungi isolated from food sources [17]. Furthermore, *L. pedunculata* EO has been shown to possess a strong cytotoxic and antiproliferative potential by inducing apoptosis in a dose-dependent manner [18,19], good acaricidal properties against *Hyalomma aegyptium*, a hematophagous ectoparasite belonging to the Ixodidae family [20], and antioxidant power [14].

In recent decades, the emergence of antimicrobial resistance (AMR) has become a global health concern, compromising the efficacy of conventional antibiotics and urging the need for alternative antimicrobial agents [25]. Essential oils, due to their complex chemical composition and multitarget modes of action, are gaining attention as potential candidates in the fight against resistant pathogens [26]. However, despite the growing interest, there is a clear lack of studies focused on the biological activity of the EO from specific taxa such as *L. pedunculata* subsp. *atlantica*.

To the best of our knowledge, only three papers have been published on the biological properties of the EO of *L. pedunculata* subsp. *atlantica* (Braun-Blanq.) Romo (syn. *L. atlantica*). Two of them concern the antibacterial activity, which was claimed to be due to the synergistic effect of certain minor compounds such as carvone, although Soulaimani et al. [21] detected a lower antimicrobial activity with respect to the Sayout work [22]. Laghzaoui et al. [20] investigated the acaricidal activity of the EO against *Hyalomma aegyptium* (Linnaeus), which is a hard-tick species of the Ixodidae family [20].

Therefore, the present study addresses this knowledge gap by evaluating for the first time a broad spectrum of biological activities—antimicrobial, antibiofilm, antioxidant, and anti-inflammatory—of the EO from *L. pedunculata* subsp. *atlantica*, aiming to contribute to the development of novel plant-based agents for therapeutic applications.

The bioactivities of EOs are associated with the presence of over 200 chemical constituents [27,28,29,30]. Furthermore, they have been studied for their efficacy in reducing biofilm formation and treating infections caused by both Gram-negative and Gram-positive strains, demonstrating significant potential in combating antibiotic resistance [31]. Therefore, the aim of this work is to characterize the biological activities of the EO from *L. pedunculata* subsp. *atlantica*, describing its chemical composition and investigating the antimicrobial, antibiofilm, antioxidant, and anti-inflammatory effects. In particular, the antimicrobial activity has been investigated toward bacterial strains responsible for different pathological infection states, including *Escherichia coli*, *Staphylococcus aureus*, *Streptococcus mutans*, *Streptococcus oralis*, and *Shigella sonnei*.

## 2. Results and Discussion

### 2.1. Chemical Composition of the Essential Oil (EO)

The hydrodistillation of *L. pedunculata* subsp. *atlantica* aerial parts, collected at a pre-flowering stage, gave a light yellow EO (**Lpa**). Overall, forty-two compounds were identified, representing 94.7% of total components, which are listed in Table 2 according to their retention indices on a DB-5 MS column and classified into five classes based on their chemical structures. Oxygenated hydrocarbons formed the main class, representing 68.9% of the total, with camphor (27.8%), fenchone (10.6%), and eucalyptol (8.5%). Monoterpene hydrocarbons occurred in a lesser amount (22.3%), among which camphene (10.9%) and *α*-pinene (7.4%) were the principal constituents of the class. Both oxygenated sesquiterpenes (2.0%) and sesquiterpene hydrocarbons (0.8%) were present in very low quantity.

In previous reports [20,21,22,23], the composition of EOs of *L. pedunculata* subsp. *atlantica*, always collected in Morocco but in different accessions and all at the full flowering stage, were analyzed. Their profiles were quite similar to **Lpa**, although some differences must be pointed out. In fact, in all the previously analyzed samples, oxygenated monoterpenes represented the main class, ranging between 44.6% and 78.0%, with camphor as the principal constituent but with higher values (30.8–50.4%) with respect to **Lpa**. On the other hand, a good quantity of eucalyptol (1,8-cineole) was observed in **Lpa** (8.5%), which is a metabolite practically absent in the other EOs. Furthermore, **Lpa** showed a quite low amount of sesquiterpenes (2.8%) when compared with the EOs of all the other accessions (4.9–17.4%). These differences could be attributed, in addition to the pedoclimatic conditions, to the different vegetative phases at the time of harvesting the plants.

### 2.2. Antimicrobial Properties of ***Lpa***

The EOs obtained from *Lavandula* genus plants have shown potent antimicrobial activities, and the main active components were camphor and fenchone [20,21,22,23]. These EOs showed significant inhibitory effects on both sensitive and resistant bacterial strains and showed strong synergistic effects when combined with the individual characteristic components of the EO, such as terpenoids [23]. It was shown how the punctual composition in terpenoids content is related to antimicrobial activity. For example, the activity can be positively affected by the presence of minor compounds such as carvone, terpinen-4-ol, and 1,8-cineole, highlighting a potential synergistic effect [23]. These data highlight that the antimicrobial potential of EOs is deeply dependent on chemical components and the way in which they interact with each other. Bouazama et al. [13] also studied the antimicrobial effects of plants such as *L. pedunculata* and *L. dentata*, highlighting that the EOs were characterized by a high percentage of oxygenated monoterpenes, including camphor. Both showed significant antibacterial activity against Gram-positive and Gram-negative strains, although they exhibited lower MICs against Gram-positive strains that were therefore more sensitive.

**Figure 2:**
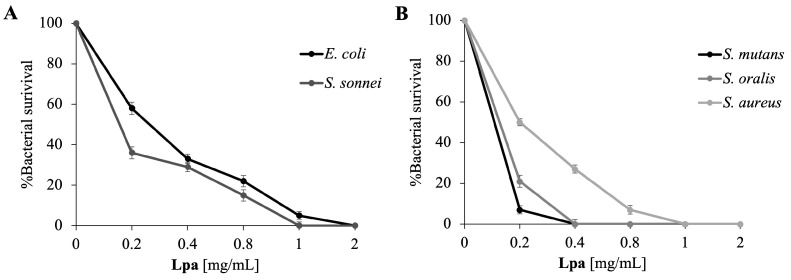
Determination of the **Lpa** antimicrobial activity at different concentrations evaluated by colony-counting assay against Gram-negative (**Panel A**) and Gram-positive (**Panel B**) strains. % Bacterial survival is represented on the *y*-axis obtained from the ratio of colony counts of treated and control. The assays were performed in three biological replicates; standard deviations are always less than 10%.

In this study, we evaluated the antimicrobial potential of the EO obtained from the aerial parts of *L. pedunculata* subsp. *atlantica*, collected at a pre-flowering stage, against Gram-positive and Gram-negative bacteria by means of a viable count assay. The dose–response curves (Figure 2) show the progression of antimicrobial activity with the increase in **Lpa** concentration. Significant bacterial targets were selected, including pathologically relevant Gram-negative strains (*E. coli* and *S. sonnei*), implicated in intestinal infections, and Gram-positive strains (*S. aureus*, *S. mutans*, and *S. oralis*) implicated in oral and skin infections. As it can be seen in Figure 2, there is a close correlation between the increase in **Lpa** concentration and the decrease in bacterial survival. In general, **Lpa** appears to be active at lower concentrations on Gram-positive bacteria than on Gram-negative bacteria. This result is in accordance with a set of recently reported data [32].

According to the previous results, it was decided to conduct further analyses on the antimicrobial effect of this promising **Lpa**, and three independent experiments were performed to determine the MIC values.

As shown in Table 3, the lowest MIC values were observed against Gram-positive bacteria, ranging from 0.2 mg/mL for *S. mutans*, the most sensitive bacterium, to 1 mg/mL for *S. aureus*. Against Gram-negative bacteria, MIC values ranged between 1 and 2 mg/mL.

These results are particularly interesting and promising, especially compared to the study by Walasek-Janusz et al. [33], which reports higher MIC values (ranging from 2.5 to 10 mg/mL) for EOs from various *Lavandula* species active against both Gram-positive and Gram-negative bacteria. Specifically, EOs of *L. angustifolia* and *L*. × *intermedia* showed higher MIC values than those observed in this study. In fact, *S. aureus* had MIC values of 10 and 5 mg/mL, respectively. Similarly, the MIC values for Gram-negative bacteria *E. coli* and *S. Typhimurium* were also higher, exhibiting values of 10 mg/mL.

### 2.3. **Lpa** Target Determination in Bacteria

**Figure 3:**
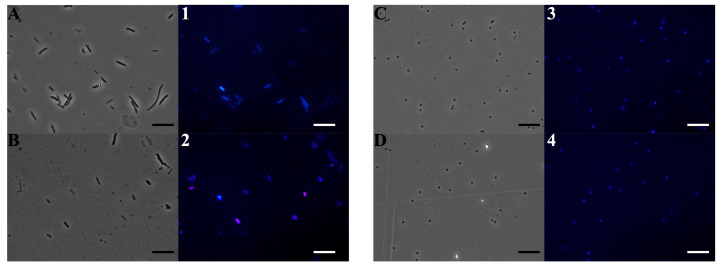
Evaluation of the antimicrobial action mechanism of **Lpa** by fluorescence microscopy. Panels show *E. coli* bacterial cells (**A**,**B**,**1**,**2**) and *S. aureus* bacterial cells (**C**,**D**,**3**,**4**). Panels (**A**–**D**) show the cells observed under the optical microscope and (**1**–**4**) under the fluorescence microscope. Untreated bacterial cells (**A**,**1**,**C**,**3**); cells treated with **Lpa** (**B**,**2**,**D**,**4**). Scale bars: 5 µm.

To investigate the mechanism of action of **Lpa**, fluorescence microscopy experiments were performed. To test the effect of EOs on bacterial membrane integrity, *E. coli* and *S. aureus* cells were used and stained with DAPI, a blue-emitting DNA fluorescent dye, and propidium iodide, which emits red light. The latter can enter cells only through damaged membranes and is therefore considered an indicator of cell membrane damage. As shown in Figure 3, panels A-1 and C-3, untreated bacterial cells, used as a control, appear intact and blue due to DAPI fluorescence. In particular, after 4 h of treatment with **Lpa** against *E. coli* and *S. aureus* (1 and 0.5 mg/mL), some *E. coli* cells (Panel 2) developed a red fluorescence, suggesting membrane disruption. On the contrary, the treatment against *S. aureus* with **Lpa** did not give red fluorescence, indicating that for Gram-positive bacteria, the cellular target is different from the bacterial membrane.

This difference in the mechanism of action can be explained by considering both the composition of the EO used and the structural differences between the two types of bacteria. **Lpa** is characterized by a good relative amount of oxygenated monoterpenes, and it contains also a significant amount of eucalyptol (8.5%). Oxygenated monoterpenes are known for their ability to interact with bacterial cell membranes, altering their permeability and causing damage that can lead to cell death. These compounds interfere with the lipid component of the plasma membrane, causing alterations in permeability and the loss of intracellular material [34]. Unlike other EOs of *L. pedunculata*, in this case, oxygenated monoterpenes represent the main class with a percentage of 68.9%, while in other studies, these compounds range between 44.6% and 78.0%. Furthermore, camphor is the main constituent although in lower concentrations than other reported EOs (30.8–50.4%) [20,21,22,23]. Another significant difference is the presence of eucalyptol (1,8-cineole) in **Lpa** (8.5%), which is a metabolite virtually absent in the EOs taxa. Finally, **Lpa** shows a rather low amount of sesquiterpenes (2.8%) compared to the other accessions analyzed (4.9–17.4%). The antimicrobial activity of **Lpa** is influenced both by its chemical composition and by the structure of the bacterial wall of the target microorganisms. Gram-negative bacteria, such as *E. coli*, have an outer membrane made of lipopolysaccharides, which acts as a protective barrier but is also particularly vulnerable to lipophilic substances such as monoterpenes. Treatment with **Lpa** can destabilize this outer membrane, allowing bioactive compounds to reach the inner cytoplasmic membrane and compromise its integrity. The resulting damage allows propidium iodide (PI) to penetrate the cell and bind to DNA, resulting in the red coloration observed.

In contrast, *S. aureus*, a Gram-positive bacterium, has a thicker cell wall, composed primarily of peptidoglycan, which provides greater protection against the direct action of oxygenated monoterpenes. Although these compounds can penetrate the cell wall and interfere with essential intracellular processes, such as protein synthesis and enzyme function, the cytoplasmic membrane is less compromised than that of Gram-negative bacteria. Consequently, PI is unable to penetrate and stain the DNA of *S. aureus* cells, which appears blue when stained with DAPI. Maybe the key role in the antimicrobial action of **Lpa** may be attributed to eucalyptol, which is a significant component of it. Eucalyptol is an oxygenated monoterpene with known antibacterial properties, and it is particularly effective against Gram-negative bacteria due to its ability to interact with cell membranes and increase their permeability [35]. This could explain why *E. coli* cells are red: eucalyptol, together with other oxygenated monoterpenes present in the EO, contributes to destabilizing the external membrane, facilitating the passage of propidium iodide and signaling irreversible damage. In fact, Fahad et al. [36], studying EOs of different eucalyptus species, whose main component was 1,8-cineole, showed that according to MIC/MBC values, Gram-negative bacteria were, in general, more sensitive to eucalyptus oils than Gram-positive bacteria. They explain that this susceptibility of Gram-negative bacteria may be due to the presence and synergism of other components of EO such as *p*-cymene, terpinolene, and 1,8-cineole, which can cause lipopolysaccharide discharge from the Gram-negative outer membrane and increase the permeability of the cytoplasmic membrane [37].

In Gram-positive bacteria such as *S. aureus*, however, the thick peptidoglycan wall may limit the direct action of eucalyptol on the cytoplasmic membrane. However, eucalyptol may still exert an antimicrobial effect by interfering with cellular metabolism and the function of intracellular enzymes in cooperation with other **Lpa** components although without causing an immediate loss of membrane integrity.

### 2.4. Antibiofilm Activity of ***Lpa***

**Figure 4:**
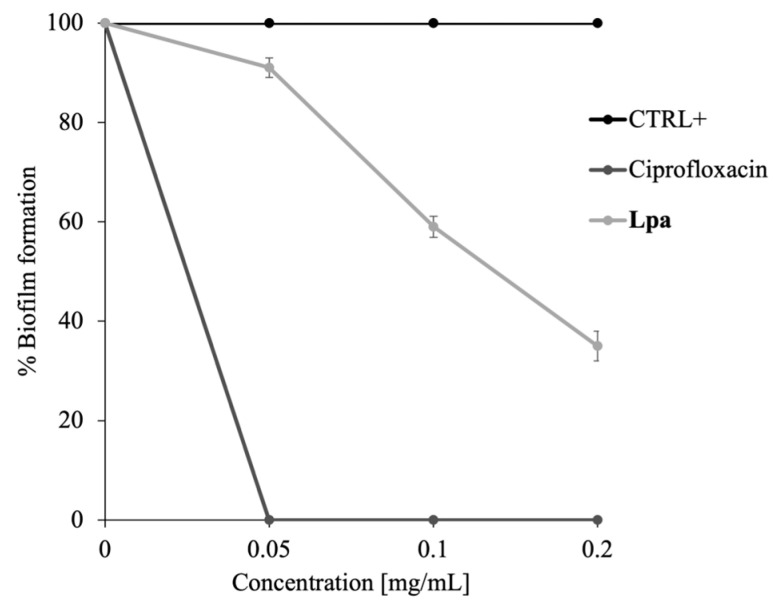
Colorimetric assay to evaluate the % of multispecies biofilm formation formed by *S. mutans* and *S. oralis* at different concentrations of **Lpa**. The positive control is represented by untreated cells and the negative control by ciprofloxacin. The assays were performed in three biological replicates; the standard deviations are always less than 10%.

**Lpa** used in low concentrations may be active in preventing biofilm formation. As is known from previous studies, several EOs [38], even at low concentrations, can have an antibiofilm effect. To validate this hypothesis, experiments were performed on multispecies biofilms of *S. oralis* and *S. mutans*. Considering that the bacterial survival % of the two strains co-incubated with 0.2 mg/mL of **Lpa** is 99.8%, the formation of multispecies biofilms with and without **Lpa** was initiated at concentrations ranging from 0.05 to 0.2 mg/mL. As can be seen in Figure 4, an inhibition of biofilm formation of about 60% occurs using **Lpa** at 0.2 mg/mL. This result demonstrates that **Lpa** can inhibit oral biofilm formation.

Indeed, the failure of conventional antibiotic treatments suggests that the eradication of microbial biofilms needs continuous updating [39]. Natural antibiofilm substances target persistent biofilms and promote the diffusion of antimicrobials into the biofilm matrix. Typically, these natural agents are active at different stages of biofilm formation to degenerate the matrix and finally kill the released cells. Indeed, Kavanaugh et al. demonstrated that certain selected essential oils—such as cassia oil, lavender oil, clove oil, and red thyme oil—can eradicate biofilms formed by *Pseudomonas* spp. and *Staphylococcus aureus* with greater efficiency than certain antibiotics, such as colistin. They highlight that essential oils act by damaging the cell wall and membrane, leading to cell lysis and the leakage of cellular contents. Furthermore, they show that these oils are effective in disrupting the biofilm matrix, thereby facilitating the elimination of bacterial cells [40]. The goal of an antibiofilm agent is to destroy the biofilm and kill the bacterial cells. The results obtained in this work demonstrate the efficacy of **Lpa** for this purpose.

### 2.5. Antioxidant Activity of ***Lpa***

**Figure 5:**
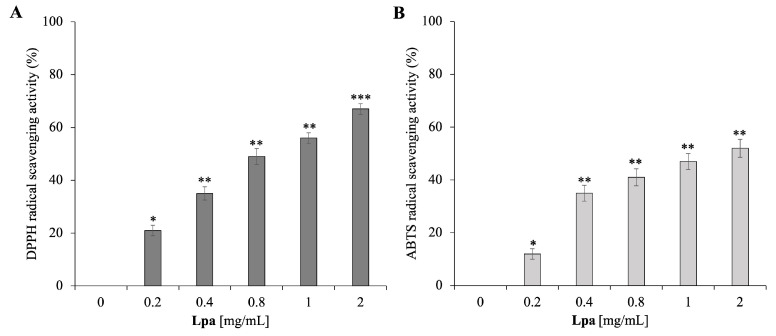
Determination of antioxidant activity of **Lpa**. Panel (**A**) shows the DPPH radical scavenging activity obtained after 30 min of incubation and reported as % of DPPH removed relative to the control. Panel (**B**) shows the ABTS scavenging activity, measured after 10 min of incubation and reported as % of ABTS removed relative to the control. Data were presented as the mean of three independent experiments. Statistical analysis was calculated using a two-tailed paired *t* test (* *p* < 0.05, ** *p* < 0.01; *** *p* < 0.001).

A reported investigation on the EO antioxidant activity [41] has shown that the scavenging ability of DPPH and ABTS radicals is also closely related to the concentration of EO and has a strong connection with its chemical composition, especially with main constituents. The primary components of **Lpa** are oxygenated terpenes, which have a great impact on antioxidant activity. According to the analysis of the primary components of EO, the antioxidant activity is positively related to the amount of oxygenated terpenoids (oxygenated monoterpenes and sesquiterpenes) [42,43]. Figure 5 shows the increasing percentage of DPPH and ABTS radical scavenging activity with increasing concentration (0–2 mg/mL) of **Lpa**. Specifically, the DPPH assay, commonly used to evaluate the ability to donate electrons and neutralize free radicals, demonstrates that **Lpa** has an effective antioxidant activity that is equal to about 60% at 2 mg/mL. Similarly, the ABTS assay, which measures the ability of the EO to neutralize highly reactive cationic radicals, confirms strong antioxidant activity, equal to 50%, suggesting that the compounds present in **Lpa** can interact with reactive oxygen species and reduce their oxidative potential. The observed scavenging activity of **Lpa** is statistically significant (*p* < 0.05), indicating that the antioxidant effects are not due to random variation but are instead consistently associated with increasing concentrations of the essential oil.

As mentioned above, this antioxidant activity can be mainly attributed to the high amount of oxygenated monoterpenes and the significant presence of eucalyptol. Oxygenated monoterpenes, including camphor and 1,8-cineole (eucalyptol), are known to possess antioxidant properties due to their ability to stabilize free radicals through electron or hydrogen transfer [44]. Furthermore, eucalyptol has been described as a compound with scavenger potential that is capable of reducing the oxidative damage induced by radical species [45]. These results are consistent with previous findings on other essential oils rich in oxygenated monoterpenes, such as rosemary and eucalyptus oils, which have also shown strong antioxidant capacities in both DPPH and ABTS assays [46,47]. In particular, the values observed for **Lpa** at 2 mg/mL are comparable to those reported for rosemary oil (55–65% DPPH inhibition at similar concentrations), highlighting its potential as a competitive natural antioxidant.

The efficacy of **Lpa** in neutralizing DPPH and ABTS radicals suggests a potential use of EO as a natural agent with antioxidant properties. Such promising results reinforce the potential application of **Lpa** in oxidative stress-related biomedical contexts, aligning with the growing body of literature that supports the role of essential oils as protective agents in cellular systems. These characteristics could be particularly useful in biomedical applications, contributing to cellular protection from oxidative stress and the inhibition of lipid peroxidation processes.

### 2.6. Cell Viability and Anti-Inflammatory Properties of ***Lpa***

**Figure 6:**
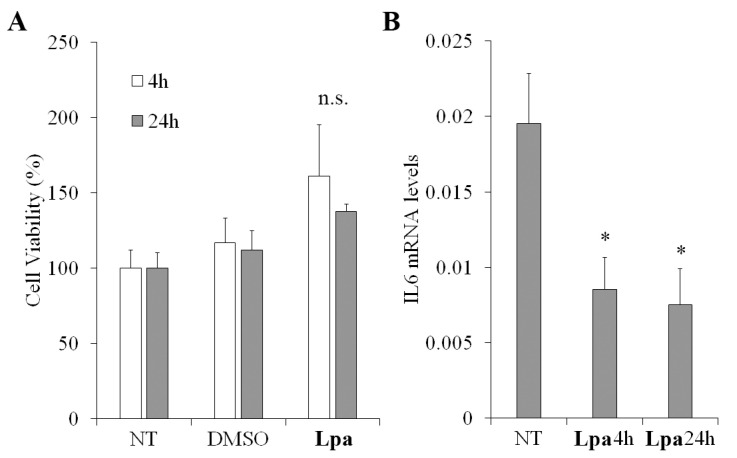
Determination of **Lpa** activity on viability and inflammation. Panel (**A**): viability of HaCat cells was assessed by the MTT assay. The untreated cells were assumed as 100%. Percent of cell viability upon 4 and 24 h of EO treatment was calculated by comparing with the untreated cells. Panel (**B**): mRNA expression levels of IL6 were assessed by real-time RT-qPCR. IL6 expression was quantified in untreated HaCat cells (NT) and upon 4 or 24 h of EO treatment and normalized by comparing to RPLP0 expression. Data were presented as the mean of three independent experiments. Statistical analysis was calculated using a two-tailed paired *t* test (* *p* < 0.05; n.s.: not significant). Error bars denote SD.

Due to the great number of constituents, EOs seem to have several potential cellular effects [48], including cell viability [49] and anti-inflammatory properties [50]. To evaluate the EO activity on cell viability, MTT assay was performed in human keratinocytes. The viability of cells increased, although not significantly, after both 4 and 24 h of treatment with EO compared to the control groups (Figure 6A). These findings were consistent with the decreased mRNA expression levels of the pro-inflammatory cytokine IL6 observed in HaCat upon 4 or 24 h of treatment with EO (Figure 6B). The expression levels of cytokine IL6, which is a well-known mediator in the inflammation process, are often investigated in anti-inflammatory assays when screening oils and compounds for therapeutic properties, as reported in previous anti-inflammatory studies on essential oils from plant [44,51,52,53].

## 3. Materials and Methods

### 3.1. Plant Material

The pre-flowering aerial parts of *L. pedunculata* subsp. *atlantica* were collected on road N 9, from Marrakech to Ouarzazate (Morocco), near Ait Ben Ammar (31°22′12″ N, 7°23′30″ O, 1555 m s/L), on a siliceous soil, in May 2023, and a voucher specimen has been deposited in STEBICEF Department, University of Palermo (PAL109773).

### 3.2. Isolation of ***Lpa***

Fresh samples were ground in a Waring blender and then subjected to hydrodistillation for 3 h, according to the standard procedure described in the *European Pharmacopoeia* [54]. The EO was dried over anhydrous sodium sulfate and stored in a sealed vial under N_2_, at −20 °C, ready for the GC-MS analysis; the sample yielded 0.3% of EO (*w*/*w*).

### 3.3. GC-MS Analysis

Chemical analysis of EO was performed on a ShimadzuQP 2010 plus equipped with an AOC-20i autoinjector (Shimadzu, Kyoto, Japan) gas chromatograph featuring a capillary column (DB-5 MS) 30 m × 0.25 mm i.d., film thickness 0.25 μm and a data processor. The oven temperature program was the following: 5 min at 40 °C, subsequently 2 °C/min up to 260 °C, then isothermal for 20 min. Injector and detector temperatures were 250 and 280 °C, respectively. He was used as the carrier gas at a flow rate of 1 mL/min. The split ratio, 1:50; acquisition mass range, *m*/*z* 40–400. All mass spectra were acquired in electron-impact (EI) mode with an ionization voltage of 70 eV. The GC conditions were the same as those reported for GC–MS analysis. The pressure was kept constant at 35 kPa. The carrier gas was He. The injection volume was 1.0 µL. The split ratio was 1:50, the ionization voltage was 70 kV, and the acquisition mass range was 40–400 *m*/*z*. The percentage in Table 2 is calculated with the TIC from MS. The settings were as follows: ionization voltage, 70 eV; electron multiplier energy 2000 V; transfer line temperature, 295 °C; solvent delay, 3 min. Linear retention indices (LRI) were determined by using retention times of *n*-alkanes (C_8_–C_40_), and the peaks were identified by comparison with mass spectra and by comparison of their relative retention indices with WILEY275, NIST 17, ADAMS, and FFNSC2 libraries.

### 3.4. Bacterial Strains

The microbiological effect was assessed using the following bacterial strains: Gram-negative: *Escherichia coli* DH5α and *Shigella sonnei* ATCC25931; and Gram-positive: *Staphylococcus aureus* ATCC6538P, *Streptococcus oralis* CECT 8313 and *Streptococcus mutans* ATCC 35668.

### 3.5. Antimicrobial Assay

To evaluate the antimicrobial activity of **Lpa** against Gram-positive and Gram-negative strains, a cell viability counting assay was used. Microbial cells were incubated with and without **Lpa** at concentrations ranging from 0.2 to 2 mg/mL and incubated for 4 h at 37 °C. Samples were then diluted and plated on agar Petri dishes. Microbial cells without **Lpa** served as positive control; in contrast, cells treated with 70% DMSO served as a negative control, since DMSO was used to resuspend the EO. The next day, the survival rate of bacterial cells was calculated by counting single colonies and comparing them to the positive control [30]. All experiments were performed in triplicate, and the results reported are the means of three independent experiments.

### 3.6. Determination of Minimal Inhibitory Concentration

Minimal inhibitory concentrations (MICs) of **Lpa** against the Gram-positive and Gram-negative strains were determined according to the microdilution method established by the Clinical and Laboratory Standards Institute (CLSI). First, 5 × 10^5^ CFU/mL was added to 95 µL of Mueller–Hinton broth (CAM-HB; Difco) supplemented or not with various concentrations (0.1–3 mg/mL) of EO. After overnight incubation at 37 °C, MIC values were determined to be the lowest concentration responsible for the lack of bacterial growth by reading the OD at 600 nm. All experiments were conducted in triplicate, and the results were presented as the average of three independent trials.

### 3.7. Fluorescence Microscopy Experiments: DAPI/PI

For the fluorescence microscopy experiments, two dyes were used: DAPI (4′,6-diamidino-2-phenylindole dihydrochloride; Sigma Aldrich, Milan, Italy) and IP (propidium iodide; Sigma Aldrich, Milan, Italy). Briefly, 100 µL of bacterial cultures of *E. coli* and *S. aureus* model strains was incubated in the dark for 4 h at 37 °C with shaking, with or without **Lpa** at sub-MIC concentration (1 and 0.5 mg/mL). After incubation, 10 µL of the bacterial culture was mixed with a solution of DAPI [1 µg/mL] and PI [20 µg/mL]. Samples were examined with an Olympus BX51 fluorescence microscope (Olympus, Tokyo, Japan) equipped with a DAPI filter (excitation/emission: 358/461 nm). Standard acquisition times for DAPI/PI dual staining were set at 1000 ms. Images were acquired using an Olympus DP70 digital camera, following the method described by Castagliuolo et al. [55].

### 3.8. Antibiofilm Tests

The antibiofilm activity of **Lpa** was evaluated against a multispecies biofilm of *S. oralis* and *S. mutans* using colorimetric tests. After determining that the minimum inhibitory concentration (MIC) of **Lpa** was higher during the co-incubation of both bacterial strains, a concentration range of 0.05 to 0.2 mg/mL was selected for further testing. Untreated microbial cells served as positive control, while those treated with ciprofloxacin (2 µg/mL) served as the negative control. A 24-well plate was incubated at 37 °C for 72 h. To evaluate antibiofilm activity, biofilms were stained with crystal violet dye after incubation. The optical density (OD) of the stained biofilm was measured at 570 nm using a Multiskan microplate reader (Thermo Electron Corporation, Waltham, MA, USA) following established protocols [29]. The percentage of biofilm formation was calculated by comparing the OD values of the peptide-treated samples to those of the untreated samples.

### 3.9. DPPH and ABTS Scavenging Capacity Assay

The measurement of DPPH and ABTS radical scavenging activity was conducted according to Napolitano et al. [56]. Different concentrations of **Lpa** (0–2 mg/mL) were added to a final volume of 1 mL. For the DPPH assay, 100% methanol containing 0.1 mM freshly prepared DPPH was used, resulting in an absorbance of ≤1.0. For the ABTS assay, 1 mL of ABTS solution (7 mM ABTS and 2.45 mM potassium persulfate) was diluted with PBS, achieving an absorbance of 0.72. The DPPH reaction was allowed to proceed for a maximum of 30 min, while the ABTS reaction was carried out for 10 min at room temperature. In the first case, the absorbance was measured at 517 nm, and in the second case, it was measured at 734 nm. The free radical scavenging activity was calculated using the following equation:

DPPH/ABTS radical scavenging activity (%) = (1 − AS/AC) × 100

where AS is the absorbance of the reacted mixture of DPPH or ABTS with the **Lpa** sample, and AC is the absorbance of the DPPH or ABTS solution.

Ascorbic acid was used as a positive control, following Napolitano et al. [56], in order to compare the antioxidant activity of the sample with that of a well-known reference antioxidant.

### 3.10. Cell Viability Assay

The MTT-based cytotoxicity assay was performed in HaCat cells. First, 20,000 cells were plated in 96-well microplates and incubated for 24 h at 37 °C, 5% CO_2_. EO was added at a concentration of 0.2 mg/mL at 4 and 24 h before the measure of the absorbance (570 nm) using a Synergy H4 Hybrid Microplate reader (Agilent, Santa Clara, CA, USA).

### 3.11. RNA Isolation and Real Time RT-qPCR

Total RNA was extracted using TRIzol reagent (Thermo Fisher Scientific, Waltham, MA, USA), and cDNA was synthesized using a LunaScript RT SuperMix Kit (New England Biolabs, Ipswich, MA, USA). Real-time RT-qPCR was performed using the Luna^®^ Universal qPCR Master Mix (New England Biolabs) in a QuantStudio™ 5 Real-Time PCR (Applied Biosystems, Waltham, MA, USA). Interleukin 6 (IL6) expression was quantified using specific oligonucleotide primers (forward: 5′- -3′; reverse: 5′- -3′) and normalized for RPLP0 expression (forward: 5′-GACGGATTACACCTTCCCACTT-3′; reverse: 5′-GGCAGATGGATCAGCCAAGA-3′).

## 4. Conclusions

Essential oil of *Lavandula pedunculata* subsp. *atlantica* (**Lpa**) shows a series of very interesting biological properties: a significant antibacterial activity against Gram-positive and Gram-negative strains as demonstrated by the significant MIC values observed toward the bacterial species *E. coli*, *S. sonnei*, *S. aureus*, *S. mutans* and *S. oralis.* The antibacterial properties of **Lpa** on the last two species are also related to a good inhibitory activity on oral biofilm formation that offers a potential for interesting biomedical application in the context of oral health. Further, the efficacy of **Lpa** in neutralizing DPPH and ABTS radicals suggests a potential use of the essential oil as a natural agent with antioxidant properties. The decreased of pro-inflammatory cytokine IL6 level observed in HaCat upon 4 or 24 h of treatment with **Lpa** is of fundamental importance in the inhibition of inflammatory processes. These characteristics could prove particularly useful in biomedical applications, contributing to the fight against bacterial infections, to the cellular protection from oxidative stress and to the prevention of the inflammatory state of human cells.

## Figures and Tables

**Table 1:** Main components (>2%) and chemical classes of the essential oils (EOs) obtained from aerial parts of *Lavandula pedunculata* subspecies.

Taxa	Origin	Compounds	MH	OM	SH	OS	O	Ref.
*L. pedunculata*	Morocco, Tafraout	camphor (53.1), eucalyptol (6.5), camphene (6.1), α-pinene (2.0)	11.7	72.7	0.5	2.1	-	[13]
*L. pedunculata*	Morocco, Khenifra	camphor (44.2), 1-*epi*-cubenol (8.6), fenchone (7.5), camphene (6.4), α-pinene (4.9)	16.0	64.1	1.6	12.4	-	[14]
*L. pedunculata*	Morocco, Khenifra	camphor (47.5), fenchone (27.1), 1-*epi*-cubenol (2.8), verbenone (2.4), borneol (2.0)	0.7	87.3	0.3	4.4	1.4	[15]
*L. pedunculata*	Morocco, Azrou	camphor (41.1), fenchone (15.8), 1,10-*diepi*-cubenol (7.5), borneol (5.7), camphene (5.8), *α*-pinene (2.1)	10.1	72.7	0.8	16.4	-	[16]
*L. pedunculata*	Portugal, Serra da Malcata	fenchone (50.5), camphor (30.0), α-pinene (7.0), limonene (2.1)	11.4	85.5	0.3	1.5	-	[17]
*L. pedunculata*	Portugal, Coa Valley	camphor (39.0), α-pinene (6.9), bornyl acetate (5.9), fenchone (5.4), camphene (4.0), *endo*-borneol (2.4)	14.7	73.0	0.8	7.3	-	[18]
*L. pedunculata*	Portugal, Coimbra	fenchone (45.5), camphor (8.7), α-pinene (8.0), eucalyptol (5.1), bornyl acetate (3.5), *α*-cadinol (2.5)	15.6	72.1	1.1	2.7	-	[19]
*L. pedunculata*	Portugal, Bragança	eucalyptol (34.3), camphor (9.9), *β*-pinene (9.0), fenchone (7.6), linalool (3.8), borneol (3.4), *α*-cadinol (3.1), *cis*-verbenol (2.8), *α*-pinene (2.5)	15.3	72.4	2.0	3.7	-	[19]
*L. pedunculata*	Portugal, Guarda	camphor (34.0), eucalyptol (25.1), fenchone (6.2), camphene (6.1), *α*-pinene (3.8), *trans*-verbenol (2.0)	15.6	75.1	0.6	0.3	-	[19]
*L. pedunculata* ssp. *atlantica*	Morocco, Touflihte	camphor (30.8), α-pinene (14.8), camphene (14.6), 1,10-di-*epi*- cubenol (11.9), fenchone (7.5), selina-3.7(11)-diene (2.9), linalool (2.8), limonene (2.1)	34.4	44.6	5.5	11.9	-	[20]
*L. pedunculata* ssp. *atlantica*	Morocco, Touflihte	camphor (41.5), fenchone (16.8), camphene (11.5), *epi*-cubenol (2.9), α-pinene (2.5), eucalyptol (2.3), *endo*-borneol (2.0)	15.8	67.5	3.0	4.5	5.8	[21]
*L. pedunculata* ssp. *atlantica*	Morocco, Tazakka	camphor (50.4), fenchone (14.1), camphene (5.6), *α*-pinene (2.4), borneol (2.3)	10.7	78.0	0.5	4.4	-	[22]
*L. pedunculata* ssp. *atlantica*	Morocco, Oulmès	camphor (39.2), fenchone (9.2), camphene (6.7), *α*-pinene (6.5), borneol (2.5), linalool (2.5), *α*-selinene (2.2), *δ*-cadinene (2.2)	16.0	55.7	8.0	9.0	-	[23]
*L. pedunculata* ssp. *lusitanica*	Portugal, Algarve	camphor (40.6), fenchone (38.0), α-fenchol (2.6), linalool (2.0)	0.1	90.6	1.6	2.0	0.6	[12]
*L. pedunculata* ssp. *lusitanica*	Portugal, Faro	fenchone (41.9), camphor (34.6), α-pinene (2.8), linalool (2.7)	5.7	86.8	0.5	0.3	0.2	[24]

MH = monoterpene hydrocarbon; OM = oxygenated monoterpene; SH = sesquiterpene hydrocarbon; OS = oxygenated sesquiterpene; O = other compound.

**Table 2:** Chemical composition of the **Lpa** aerial parts collected in Morocco.

No.	Compounds ^a^	LRI ^b^	LRI ^c^	Area (%) ^d^
1	Tricyclene	924	926	0.7
2	*α*-Pinene	935	938	7.4
3	Camphene	947	947	10.9
4	*β*-Pinene	972	975	0.6
5	1-Octen-3-ol	979	979	0.6
6	*β*-Myrcene	990	988	0.4
7	*α*-Terpinene	1011	1015	0.2
8	*p*-Cymene	1019	1021	1.0
9	Eucalyptol	1025	1031	8.5
10	*β*-*cis*-Ocimene	1035	1032	0.6
11	*β*-*trans*-Ocimene	1042	1045	0.2
12	*γ*-Terpinene	1054	1056	0.3
13	*trans*-Linalool oxide	1069	1072	0.3
14	Fenchone	1080	1078	10.6
15	*β*-Linalool	1099	1103	3.1
16	Fenchol	1110	1110	0.5
17	1,7,7-Trimethylbicyclo[2.2.1]hept-5-en-2-ol	1118	1115	0.5
18	Camphor	1136	1139	27.8
19	Pinocarvone	1156	1154	0.2
20	Borneol	1157	1157	1.5
21	*α*-Phellandren-8-ol	1159	1159	0.8
22	4-Terpineol	1169	1172	1.0
23	4-Methylacetophenone	1173	1175	0.1
24	*p*-Cymen-8-ol	1177	1179	0.2
25	*α*-Terpineol	1183	1180	0.5
26	Myrtenal	1185	1188	0.4
27	Myrtenol	1189	1191	2.1
28	Verbenone	1197	1201	0.3
29	*cis*-Carveol	1210	1214	0.2
30	Fenchyl acetate	1212	1217	0.3
31	Carvone	1233	1231	0.4
32	Bornyl acetate	1278	1283	6.7
33	Myrtenyl acetate	1317	1316	2.8
34	α-Cubebene	1342	1345	*t*
35	Eugenol	1347	1348	0.1
36	Neryl acetate	1367	1362	0.1
37	Caryophyllene	1409	1413	0.2
38	*δ*-Cadinene	1514	1519	0.6
39	Viridiflorol	1579	1580	0.6
40	Di-epi-1,10-cubenol	1589	1587	0.5
41	*δ*-Cadinol	1628	1626	0.4
42	*τ*-Cadinol	1640	1640	0.5
	**Monoterpene Hydrocarbons**			**22.3**
	**Oxygenated Monoterpenes**			**68.9**
	**Sesquiterpene Hydrocarbons**			**0.8**
	**Oxygenated Sesquiterpenes**			**2.0**
	**Others**			**0.7**
	**Total**			**94.7**

^a^ Compounds are classified in order of linear retention index of non-polar column DB-5MS. ^b^ LRI calculated for DB-5MS non-polar column; ^c^ Linear retention indices based on literature https://webbook.nist.gov/; accessed on 6 February 2025); ^d^ content is the peak volume percentage of compounds in the EO sample; *t*: traces (<0.10).

**Table 3:** Determination of the minimum inhibitory concentration values of bacterial growth (MIC expressed as mg/mL) of **Lpa** against Gram-negative and Gram-positive bacteria. The values were obtained from a minimum of three biological replicates.

Strains	MIC [mg/mL]
*E. coli*	2
*S. sonnei*	1
*S. aureus*	1
*S. mutans*	0.2
*S. oralis*	0.4

## Data Availability

The original contributions presented in this study are included in the article. Further inquiries can be directed to the corresponding author.
